# Supplementary material for: MetaRibo-Seq measures translation in microbiomes
Source: Nat Commun. 2020 Jun 29;11:3268. doi: 10.1038/s41467-020-17081-z (PMC7324362; doi:10.1038/s41467-020-17081-z)
Supplement: Supplementary file 10 — Supplementary Data 7 [file 41467_2020_17081_MOESM10_ESM.zip › File2/Confidence_High_Taxonomy/200105_out.krona.html]

Javascript must be enabled to view this page.

members
magnitude
magnitudeUnassigned
count
unassigned
taxon
rank

200105\_out

86

86
superkingdom
2

86
phylum
1239

186801
class
86

4

SRS011239\_contig\_number\_contig-100\_28369.69942SRS019601\_contig\_number\_contig-100\_25385.65741SRS020233\_contig\_number\_contig-100\_42492.119783SRS045826\_contig\_number\_contig-100\_36107.36108
order
86
186802

186806
family
2

1730
2
genus

species
1
39485

SRS104084\_contig\_number\_498

142586
1
species

SRS148159\_contig\_number\_30432


SRS020869\_contig\_number\_contig-100\_15660.51386
1
family
186803
9

841
genus
2

1897015
1
species

SRS014979\_contig\_number\_35217

301301
1
species

SRS014855\_contig\_number\_11628

1898203
5
species

SRS063040\_contig\_number\_27624SRS077194\_contig\_number\_contig-100\_1490.157878SRS1041033\_contig\_number\_15712SRS104485\_contig\_number\_2947SRS893170\_contig\_number\_contig-100\_555.35195

1
genus
572511

33039
species
1

SRS049900\_contig\_number\_4544

31979
family
68

genus
68
1485


SRS1041038\_contig\_number\_4717SRS893373\_contig\_number\_1364
2
species
1896990

2
species
2293016

SRS016095\_contig\_number\_contig-100\_1305.171861SRS1041140\_contig\_number\_13045

species
64
1262828

SRS011134\_contig\_number\_51429SRS012273\_contig\_number\_contig-100\_8666.164947SRS013476\_contig\_number\_contig-100\_19249.123643SRS013951\_contig\_number\_1103SRS014613\_contig\_number\_contig-100\_423.111804SRS015431\_contig\_number\_42344SRS015578\_contig\_number\_contig-100\_5612.150370SRS015579\_contig\_number\_22605SRS015663\_contig\_number\_6334SRS015694\_contig\_number\_22616SRS015854\_contig\_number\_22516SRS016840\_contig\_number\_contig-100\_9985.9986SRS016989\_contig\_number\_11961SRS017307\_contig\_number\_contig-100\_4980.4980SRS017821\_contig\_number\_26092SRS018427\_contig\_number\_26072SRS018836\_contig\_number\_contig-100\_4383.94938SRS019068\_contig\_number\_72962SRS019161\_contig\_number\_contig-100\_16.222873SRS019267\_contig\_number\_8446SRS019496\_contig\_number\_contig-100\_15455.15456SRS019693\_contig\_number\_contig-100\_13661.56086SRS021948\_contig\_number\_6034SRS022071\_contig\_number\_38856SRS022609\_contig\_number\_44784SRS024132\_contig\_number\_20552SRS049773\_contig\_number\_27642SRS051031\_contig\_number\_30232SRS051882\_contig\_number\_5741SRS052697\_contig\_number\_26310SRS053335\_contig\_number\_10899SRS054905\_contig\_number\_13415SRS063985\_contig\_number\_30650SRS064276\_contig\_number\_40948SRS075773\_contig\_number\_contig-100\_22924.104185SRS075821\_contig\_number\_15026SRS075984\_contig\_number\_22116SRS077231\_contig\_number\_contig-100\_2175.41966SRS077502\_contig\_number\_8850SRS077552\_contig\_number\_27766SRS078419\_contig\_number\_17482SRS098571\_contig\_number\_58327SRS098644\_contig\_number\_39579SRS105153\_contig\_number\_5060SRS1055022\_contig\_number\_22524SRS1055067\_contig\_number\_13830SRS142503\_contig\_number\_33338SRS142542\_contig\_number\_contig-100\_939.85270SRS143342\_contig\_number\_14757SRS143372\_contig\_number\_9616SRS143466\_contig\_number\_32172SRS143523\_contig\_number\_22472SRS143895\_contig\_number\_contig-100\_15928.179832SRS144135\_contig\_number\_9665SRS144183\_contig\_number\_30337SRS144362\_contig\_number\_31157SRS146813\_contig\_number\_771SRS147766\_contig\_number\_14025SRS147977\_contig\_number\_9587SRS148424\_contig\_number\_30684SRS149075\_contig\_number\_contig-100\_28251.28252SRS149181\_contig\_number\_30076SRS893270\_contig\_number\_contig-100\_11688.91978SRS971275\_contig\_number\_contig-100\_584.256405

541000
family
1

1
species
1898205

SRS018623\_contig\_number\_14595

216572
family
1


SRS015190\_contig\_number\_2335
459786
genus
1


SRS065504\_contig\_number\_28689
species
1
1897043
